# Correlations between circulating methylmalonic acid levels and all-cause and cause-specific mortality among patients with diabetes

**DOI:** 10.3389/fnut.2022.974938

**Published:** 2022-11-29

**Authors:** Jiao Wang, Yunliang Tang, Ying Liu, Wei Cai, Jixiong Xu

**Affiliations:** ^1^Department of Endocrinology and Metabolism, The First Affiliated Hospital of Nanchang University, Nanchang, China; ^2^Jiangxi Clinical Research Center for Endocrine and Metabolic Disease, Nanchang, China; ^3^Jiangxi Branch of National Clinical Research Center for Metabolic Disease, Nanchang, China; ^4^Department of Rehabilitation Medicine, The First Affiliated Hospital of Nanchang University, Nanchang, China; ^5^Department of Medical Genetics and Cell Biology, Medical College of Nanchang University, Nanchang, China

**Keywords:** diabetes, mortality, cardiovascular diseases, cancer, biomarkers

## Abstract

**Aims:**

Evidence regarding serum methylmalonic acid (MMA) levels and mortality in individuals with diabetes is limited. This study aimed to evaluate the correlation between MMA and all-cause and cause-specific deaths in patients with diabetes.

**Materials and methods:**

This is a population-based cohort study based on data from both the National Health and Nutrition Examination Survey (NHANES) and National Death Index from 1999 to 2014. We assessed the association of serum MMA concentrations with mortality using Cox proportional hazard models after adjusting for lifestyle, demographic factors, and comorbidities.

**Results:**

Among the 3,097 participants, 843 mortalities occurred during a median follow-up of 4.42 years. There were 242 deaths due to cardiovascular disease (CVD) and 131 cancer-associated deaths. After multivariate adjustment, elevated serum MMA levels were markedly correlated with a high risk of all-cause, CVD-, and cancer-related deaths. Each one-unit increase in the natural log-transformed MMA level correlated with increased risk of all-cause mortality (2.652 times), CVD mortality risk (3.153 times), and cancer-related mortality risk (4.514). Hazard ratios (95% confidence intervals [CIs]) after comparing participants with MMA < 120 and ≥250 nmol/L were 2.177 (1.421–3.336) for all-cause mortality, 3.560 (1.809–7.004) for CVD mortality, and 4.244 (1.537–11.721) for cancer mortality.

**Conclusion:**

Higher serum MMA levels were significantly associated with higher all-cause, CVD, and cancer mortality. These findings suggest that maintaining lower MMA status may lower mortality risk in individuals with diabetes.

## Introduction

The worldwide incidence and prevalence of diabetes are continuously increasing; approximately 592 million people are believed to have diabetes by 2,035 (1). Diabetes is correlated with increased risk of cardiovascular disease (CVD) and is a risk factor for various tumors including liver, pancreatic, breast, colon, and endometrial cancer (2–5). Identifying diabetes-related modifiable factors is significant to prevent or delay diabetes complications and early death.

Methylmalonic acid (MMA) is a by-product of propionate catabolism, in which propionyl-CoA generated from the catabolism of branched-chain amino acids, cholesterol, and odd-chain fatty acids is transformed to methylmalonyl-CoA in the mitochondria (6). Vitamin B12, an important co-factor of L-methylmalonyl-CoA mutase, is involved in the conversion of methylmalonyl-CoA to succinyl-CoA (7). Deficiency of vitamin B12 leads to an accumulation of MMA due to inactivation of mitochondrial L-methylmalonyl-CoA mutase (8). For this reason, MMA is acknowledged as a biomarker of vitamin B12 deficiency, wherein the MMA concentration is elevated.

There is increasing evidence suggesting the major role of MMA in mitochondrial dysfunctions as well as oxidative stress, partially by dysregulating the mitochondrial respiratory chain and initiating the release of reactive oxygen species (ROS) (9, 10). The elevated serum MMA levels are in the range of >260–350 nmol/L (11). Elevated MMA levels in patients with normal vitamin B12 levels have been reported in various conditions (such as old age and iron deficiency) and diseases (such as renal insufficiency, heart failure, and diabetes) that correlate with oxidative stress, especially in patients with several comorbidities (12). Thus, while circulating MMA levels partly depend on plasma vitamin B12, they might also reflect different conditions and diseases. Therefore, the clinical utility of this marker should be carefully considered in different patients.

Mitochondrial dysfunction is known to contribute to the progression of diabetes and diabetes-related complications (13–15). Since MMA metabolism relies on healthy mitochondria, serum MMA may be a favorable potential marker for predicting adverse clinical outcomes in chronic diseases. However, the prognostic significance of MMA in diabetes is yet to be elucidated. Therefore, in this study, we prospectively assessed the correlations between serum MMA levels and cause-specific and all-cause mortality in a nationally representative sample of adult patients with diabetes in the United States.

## Materials and methods

### Study population

The National Health and Nutrition Examination Survey (NHANES), performed by the Centers for Disease Control and Prevention (CDC), is designed to enroll an illustrative sample of non-institutionalized civilians in the United States and is released every 2 years. The survey involved in-home interviews, physical examinations, and collection of biological samples at mobile examination centers. The survey plan and study protocol were approved by the ethical review board of the National Center for Health Statistics, and all participants provided written informed consent.

**FIGURE 1:**
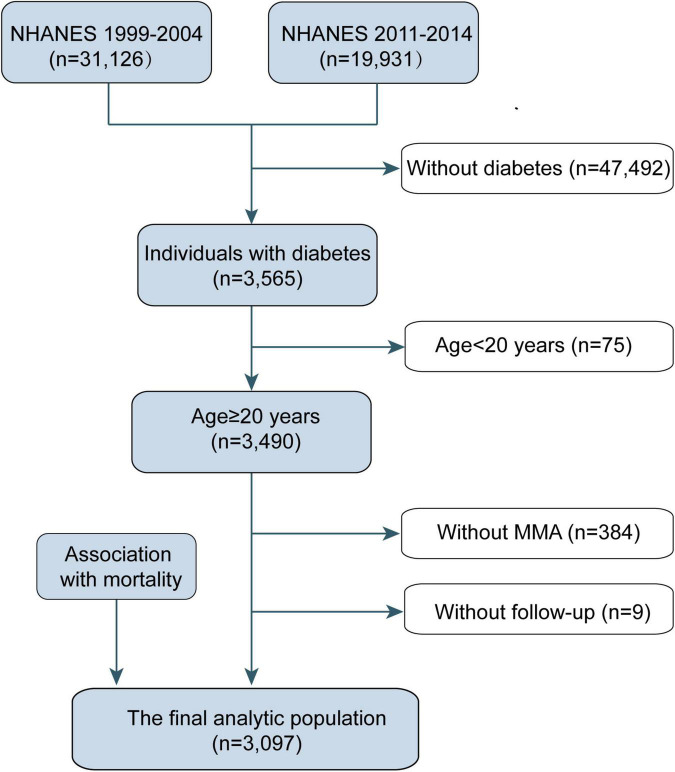
Eligible participants and those included in the analyses of the associations between circulating methylmalonic acid (MMA) levels and all-cause and cause-specific mortality in individuals with diabetes. NHANES, National Health and Nutrition Examination Survey.

For this study, we used data from five cycles of NHANES from 1999 to 2014 (MMA data were not available in NHANES 2005–2010). The study sample comprised the 51,057 participants of the NHANES cycles (1999–2000, 2001–2002, 2003–2004, 2011–2012, and 2013–2014). We excluded participants without diabetes (*n* = 47,492), those aged <20 years (*n* = 75), those with missing MMA values (*n* = 384), and those without follow-up data (*n* = 9). Ultimately, 3,097 participants were included in this study (Figure 1). Diabetes was defined as self-reported, diagnosed diabetes, insulin use or administration of oral hypoglycemic medication, fasting glucose levels >7.0 mmol/L, and/or blood glucose level ≥11.1 mmol/L (2 h oral glucose tolerance test).

### Measurement of serum methylmalonic acid

In the 1999–2004 surveys, serum MMA levels were assessed using gas chromatography-mass spectrometry. Since 2011, serum MMA levels were assessed using standardized liquid chromatography-tandem mass spectrometry. The detailed protocols are available on the National Health and NHANES website.

### Ascertainment of mortality

To determine the mortality status of the NHANES participants, we linked all eligible participants from the NHANES to the National Death Index with unique sequence numbers until December 31st, 2015. The leading cause of mortality was ascertained based on the International Classification of Diseases 10th revision (ICD-10). CVD-associated deaths were defined by ICD-10 codes I00–I09, I11, I13, I20–I51, or I60–I69. In total 843 deaths, including 242 due to CVD and 131 due to cancer, were documented.

### Evaluation of covariates

Information on age, race/ethnicity, education level, sex, family income, disease status, smoking status, physical activity, and medication used was obtained using questionnaires. Body mass index (BMI; the ratio of weight in kilograms to height in meters squared) was measured during physical examinations. Race/ethnicity was classified as follows: non-Hispanic White, Mexican American, non-Hispanic Black, or other. Self-reported educational status was grouped as less than high school, high school or equivalent, and college or above. Family income classification was defined as the ratio of family income to federal poverty level (≤1.0, 1.0–3.0, and >3.0). A high income-to-poverty ratio implies high family income status. Smoking status was classified as never-smoker (had smoked <100 cigarettes in their lifetime), former smoker (had smoked >100 cigarettes but did not smoke during survey time), and current smoker (had smoked >100 cigarettes and were still smoking during the time of survey). Physical activity was classified as vigorous activity (vigorous activities for >10 min, which caused heavy sweating or a large increase in heart rate or breathing over the past 30 days), moderate activity (moderate activity for ≥10 min that caused light sweating only or a slight to moderate increase in heart rate or breathing over the past 30 days), and inactivity. Medication use was classified as no insulin or pills, only diabetes pills, only insulin, and pills and insulin. Measurement of serum MMA was classified as gas chromatography-mass spectrometry and standardized liquid chromatography-tandem mass spectrometry.

In addition, plasma vitamin B12, total cholesterol, high-density lipoprotein (HDL) cholesterol, uric acid, albumin, alanine aminotransferase (ALT), and creatinine levels were measured at baseline when the participants provided their blood samples. Rigorous processes were performed throughout blood collection and analysis. The details are described in the NHANES Laboratory/Medical Technologists Procedures Manual.

### Statistical analysis

Statistical analyses accounted for the complex, stratified, multistage, and cluster-sampling design (including oversampling of certain subpopulations) of the NHANES using sample weights, strata, and primary sampling units embedded in the NHANES data. Population features collected at the NHANES enrollment examination are shown as mean ± SE for continuous variables, while categorical variables are presented as frequencies (%). Population features according to MMA quartiles were compared using ANOVA (continuous variables) or χ^2^ (categorical variables) tests.

**FIGURE 2:**
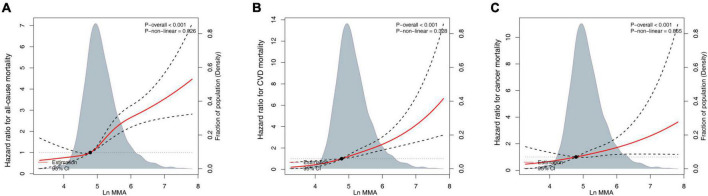
Continuous dose-response associations between methylmalonic acid (MMA) and mortality. **(A)** All-cause mortality. **(B)** Cardiovascular disease (CVD)-associated mortality. **(C)** Cancer-associated mortality. Models adjusted for age (continuous), sex (female or male), race/ethnicity (non-Hispanic White, Mexican American, non-Hispanic Black, or other), body mass index (BMI) (<25.0, 25.0–30, or ≥30 kg/m^2^), education level (less than high school, high school or equivalent, or college, and above), family income-poverty ratio (≤1.0, 1.0–3.0, or >3.0), alcohol consumption (continuous), smoking status (never-smoker, former smoker, or current smoker), physical activity (vigorous activity, moderate activity, or inactivity), vitamin B12 (pmol/L, continuous), and diabetes medication use (no insulin or pills, only diabetes pills, only insulin, or pills and insulin).

A restricted cubic spline with a multiple-adjusted Cox regression model was used to visualize the association between all-cause mortality and log-transformed MMA. We established that all-cause mortality was not markedly high when MMA was <120 nmol/L (Figure 2). There is a lack of a standard criterion for stratifying MMA levels, and previous research defined plasma MMA ≥ 250 nmol/L as a diagnostic criterion for functional vitamin B12 deficiency (10). Thus, MMA status was classified into four groups: <120, 120–175, 175–250, and ≥250 nmol/L. The association of serum MMA concentrations with CVD mortality, all-cause mortality, and cancer mortality was evaluated using Cox proportional hazards regression models with the following covariates: age, sex, race/ethnicity (non-Hispanic Black, Mexican American, non-Hispanic White, other), education levels (high school or equivalent, less than high school, college, or above), family income-poverty ratio (≤1.0, 1.0–3.0, >3.0), BMI (<25.0, 25.0–30, ≥30 kg/m^2^), alcohol consumption, smoking status (ever-smoker, never-smoker, current smoker), medication use (no insulin or pills, only diabetes pills, only insulin, pills, and insulin), physical activity (vigorous activity, moderate activity, inactivity), vitamin B12, total cholesterol, HDL-cholesterol, uric acid, albumin, ALT, creatinine, and self-reported disease.

Stratification analysis was performed for the correlation between baseline MMA and all-cause mortality in subgroups by age (≤60 and >60 years), sex (female and male), race/ethnicity (White and non-White), current smoking (no and yes), vitamin B12 (<400 and ≥400 pmol/L), and physical activity (vigorous or moderate activity and inactive). The survey-weighted Wald test was used to assess potential associations between MMA levels and stratification factors for mortality.

R (The R Foundation, Vienna, Austria)^1^ and Empower (R) (X&Y Solutions, Boston, MA, USA)^2^ statistical packages were used for analyses. Two-sided *p*-values < 0.05 denoted significance.

## Results

### Baseline characteristics

A total of 3,097 adults with diabetes (mean age: 61.8 ± 0.24 years; 51.5% male) at the exit visit were included in the final data analysis. The median follow-up duration was 53 months (range: 1–201 months). Patients with elevated MMA levels were likely to be older and non-Hispanic White, with low educational levels, family income, physical activity, vitamin B12, and total cholesterol; and higher alcohol consumption, uric acid, albumin, ALT, and creatinine levels (all *P* < 0.05). The baseline characteristics of the study participants based on their serum MMA status are presented in Table 1.

**TABLE 1:** Baseline characteristics of participants with diabetes according to serum methylmalonic acid (MMA) concentrations in National Health and Nutrition Examination Survey (NHANES).

	Total	Serum MMA concentrations, nmol/L*				
		<120	120–175	175–250	≥250	*P*-value
Number of patients	3,097	927	1012	572	586	
Age, years	61.8 ± 0.24	55.6 ± 0.43	60.9 ± 0.41	66.0 ± 0.52	68.9 ± 0.46	<0.001
Sex						0.447
Male	1,596 (51.5)	458 (49.4)	529 (52.3)	305 (53.3)	304 (51.9)	
Female	1,501(48.5)	469 (50.6)	483 (47.7)	267 (46.7)	282 (48.1)	
Race/ethnicity*						<0.001
Non-Hispanic white	1,166 (37.6)	216 (23.3)	379 (37.5)	266 (46.5)	305 (52.0)	
Non-Hispanic black	782 (25.3)	282 (30.4)	273 (27.0)	123 (21.5)	104 (17.7)	
Mexican American	660 (21.3)	252 (27.2)	219 (21.6)	90 (15.7)	99 (16.9)	
Other	489 (15.8)	177 (19.1)	141 (13.9)	93 (16.2)	78 (13.3)	
Education levels						0.003
Less than high school	1,256 (40.6)	349 (37.7)	387 (38.3)	255 (44.6)	265 (45.5)	
High school or equivalent	675 (21.8)	202 (21.8)	220 (21.8)	122 (21.3)	131 (22.5)	
College or above	1,160 (37.5)	375 (40.5)	404 (40.0)	195 (34.1)	186 (32.0)	
Family income-poverty ratio						0.001
≤1.0	726 (25.7)	205 (24.5)	233 (25.1)	143 (27.3)	145 (27.3)	
1.0–3.0	1,295 (45.9)	369 (44.1)	409 (44.0)	243 (46.4)	274 (51.6)	
>3.0	799 (28.3)	262 (31.3)	287 (30.9)	138 (26.3)	112 (21.1)	
BMI, kg/m^2^						0.122
<25.0	463 (15.5)	120 (13.2)	148 (15.0)	92 (16.8)	103 (19.0)	
25.0–30	958 (32.1)	304 (33.3)	315 (32.0)	171 (31.2)	168 (31.1)	
≥30	1,567 (52.4)	488 (53.5)	524 (53.1)	285 (52.0)	270 (49.9)	
Alcohol drinks, times/day	2.5 ± 0.06	2.7 ± 0.11	2.6 ± 0.12	2.3 ± 0.14	2.0 ± 0.12	<0.001
Smoking status						<0.001
Never smoker	1,482 (47.9)	475 (51.4)	460 (45.5)	272 (47.6)	275 (47.0)	
Ever smoker	1,093 (35.3)	261 (28.2)	362 (35.8)	233 (40.7)	237 (40.5)	
Current smoker	519 (16.8)	189 (20.4)	190 (18.8)	67 (11.7)	73 (12.5)	
Medication use						<0.001
No insulin or pills	449 (16.5)	145 (18.1)	146 (16.6)	77 (15.3)	81 (15.6)	
Only diabetes pills	1,557 (57.2)	499 (62.3)	529 (60.3)	271 (53.9)	258 (49.7)	
Only insulin	357 (13.1)	72 (9.0)	107 (12.2)	72 (14.3)	106 (20.4)	
Pills and insulin	337 (13.1)	85 (10.6)	95 (10.8)	83 (16.5)	74 (14.3)	
Physical activity						<0.001
Vigorous activity	331 (10.7)	143 (15.4)	122 (12.1)	41 (7.2)	25 (4.3)	
Moderate activity	879 (28.4)	272 (29.3)	294 (29.1)	163 (28.5)	150 (25.6)	
Inactive	1,886 (60.9)	512 (55.2)	595 (58.9)	368 (64.3)	411 (70.1)	
Vitamin B12, pmol/L	495.5 ± 25.89	636.8 ± 84.52	480.0 ± 12.77	372.7 ± 10.30	334.3 ± 8.57	<0.001
Total cholesterol, mmol/L	5.0 ± 0.02	5.1 ± 0.05	5.0 ± 0.04	4.9 ± 0.05	4.9 ± 0.06	0.012
HDL-cholesterol, mmol/L	1.2 ± 0.01	1.25 ± 0.01	1.24 ± 0.01	1.22 ± 0.01	1.21 ± 0.02	0.100
Uric acid, umol/L	339.5 ± 1.69	313.0 ± 2.8	335.4 ± 2.71	352.9 ± 3.94	374.4 ± 4.43	<0.001
Albumin, g/L	41.5 ± 0.06	42.0 ± 0.11	41.6 ± 0.10	41.3 ± 0.16	40.6 ± 0.17	<0.001
ALT, U/L	26.5 ± 0.56	28.5 ± 0.75	27.5 ± 0.63	26.6 ± 2.47	21.7 ± 0.59	<0.001
Creatinine, umol/L	91.5 ± 1.34	68.7 ± 0.69	80.3 ± 0.83	93.8 ± 1.51	144.8 ± 6.21	<0.001
Self-reported disease						−
Hypertention	1,999 (64.5)	525 (56.6)	647 (63.9)	398 (69.6)	429 (73.2)	
Hypercholesterolemia	1,653 (53.4)	482 (52.0)	535 (52.9)	312 (54.5)	324 (55.3)	

Data are the mean (standard error) or%. BMI, body mass index, ALT; glutamic pyruvic transaminase.

*Race/ethnicity was determined using preferred terminology from the National Center for Health Statistics as non-Hispanic white, non-Hispanic black, and Mexican American. Mexican American individuals were oversampled rather than broader groups of individuals from Latin America. Other includes Asian, other Hispanic, Alaskan native, and multiracial individuals.

### Relations of methylmalonic acid with mortality

**FIGURE 3:**
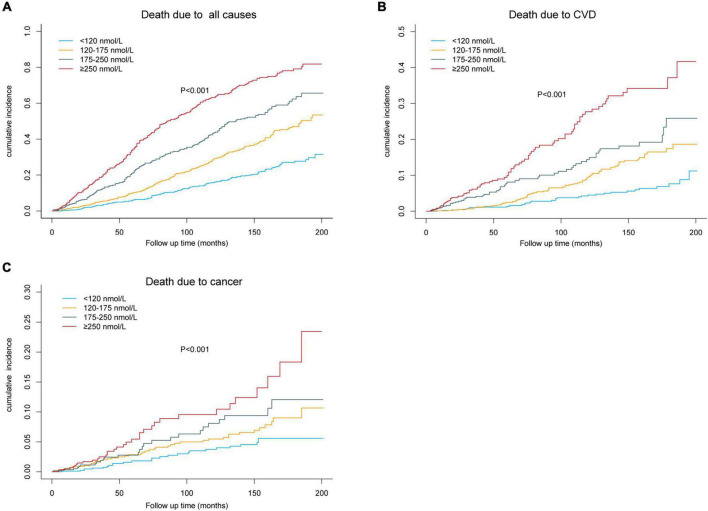
Kaplan–Meier plots for all-cause and cause-specific mortality by baseline methylmalonic acid (MMA) strata. **(A)** All-cause mortality. **(B)** Cardiovascular disease (CVD) mortality. **(C)** Cancer mortality.

In the 3,097 participants, 843 mortalities were reported during a mean follow-up of 53 months (20,966 person-years). Of them, 242 deaths were due to CVD while 131 deaths were due to cancer. After multivariate adjustment, including age, sex, race/ethnicity, BMI, education level, family income-poverty ratio, alcohol consumption, smoking status, physical activities, vitamin B12, and diabetes medication use, elevated serum MMA levels were markedly correlated with low all-cause, CVD, and cancer mortality (Table 2). Multivariate adjusted hazard ratios (HRs) and 95% confidence intervals (CIs) from lowest to highest serum MMA categories (<120, 20–175, 175–250, and ≥250 nmol/L) were 1.00 (reference), 1.228 (0.842–1.790), 1.698 (1.098–2.628), and 2.177 (1.421–3.336), respectively, for all-cause mortality (*P*_*trend*_ = 0.002); 1.00 (reference), 1.852 (1.007–3.406), 1.429 (0.666–3.067), and 3.560 (1.809–7.004), respectively, for CVD mortality (*P*_*trend*_ = 0.001); and 1.00 (reference), 1.229 (0.471–3.204), 1.996 (0.716–5.569), and 4.244 (1.537–11.721), respectively, for cancer mortality (*P*_*trend*_ = 0.015). Weighted Kaplan–Meier curves revealed marked dose-response trends across MMA strata for cause-specific and all-cause mortality, even after 10 years of follow-up, and all-cause mortality across MMA strata exhibited over-time divergence (Figure 3).

**TABLE 2:** Hazard ratio (HR) (95% confidence intervals [CIs]) for all-cause and cause-specific mortality according to serum methylmalonic acid (MMA) concentrations among patients with diabetes in National Health and Nutrition Examination Survey (NHANES).

	Serum MMA concentrations, nmol/L					
Causes of death	<120	120–175	175–250	≥250	*p*-trend	Per one-unit increment innatural log-transformed MMA
**All causes mortality**						
Number of deaths/total	131/927	252/1,012	188/572	272/586		
Crude	1.000 (ref.)	1.927 (1.559–2.380)	3.234 (2.586–4.045)	5.609 (4.546–6.921)	0.000	4.596 (3.891–5.428)
Model 1	1.000 (ref.)	1.466 (1.183–1.816)	2.005 (1.590–2.527)	3.097 (2.477–3.873)	0.000	2.977 (2.440–3.633)
Model 2	1.000 (ref.)	1.287 (0.888–1.865)	1.665 (1.082–2.561)	2.119 (1.395–3.219)	0.003	2.443 (1.472–4.053)
Model 3	1.000 (ref.)	1.225 (0.839–1.789)	1.713 (1.104–2.657)	2.181 (1.421–3.347)	0.002	2.683 (1.575–4.572)
**CVD mortality**						
Number of deaths	33	73	54	82		
Crude	1.000 (ref.)	1.933 (1.387–2.694	2.932 (2.050–4.194)	5.116 (3.663–7.145)	0.000	4.383 (3.335–5.760)
Model 1	1.000 (ref.)	1.490 (1.065–2.086)	1.851 (1.279–2.680)	2.914 (2.042–4.157)	0.000	2.902 (2.093–4.025)
Model 2	1.000 (ref.)	2.470 (1.159–5.264)	1.008 (0.359–2.827)	2.736 (1.156–6.476)	0.016	3.361 (1.663–6.796)
Model 3	1.000 (ref.)	2.268 (1.053–4.883)	0.991 (0.348–2.825)	2.509 (1.044–6.027)	0.038	2.062 (1.728–5.842)
**Cancer mortality**						
Number of deaths	26	45	26	34		
Crude	1.000 (ref.)	1.712 (1.056–2.776)	2.167 (1.256–3.738)	3.310 (1.978–5.538)	0.000	3.322 (2.037–5.419)
Model 1	1.000 (ref.)	1.394 (0.853–2.277)	1.498 (0.852–2.634)	2.057 (1.190–3.553)	0.008	2.190 (1.226–3.910)
Model 2	1.000 (ref.)	1.418 (0.564–3.564)	1.968 (0.713–5.429)	3.973 (1.532–10.301)	0.017	4.755 (1.694–13.346)
Model 3	1.000(ref.)	1.230 (0.472–3.205)	2.000 (0.717–5.578)	4.242 (1.537–11.712)	0.015	4.515 (1.462–13.947)

Model 1: adjusted for age (continuous), sex (male, or female), and race/ethnicity (non-Hispanic white, non-Hispanic black, Mexican American, or other).

Model 2: further adjusted (from Model 1) for BMI (<25.0, 25.0–30, or ≥30 kg/m^2^), education level (less than high school, high school or equivalent, or college or above), family income-poverty ratio (≤1.0, 1.0–3.0, or >3.0), Alcohol drinks (continuous), smoking status (never smoker, ever smoker, or current smoker), and Physical activity (vigorous activity, moderate activity, or Inactive).

Model 3: further adjusted (from Model 2) for Vitamin B12 (pmol/L, continuous), diabetes medication use (no insulin or pills, only diabetes pills, only insulin, or pills and insulin), Measurement of serum MMA (gas chromatography-mass spectrometry, standardized liquid chromatography-tandem mass spectrometry).

To evaluate the non-linearity of the association between MMA and mortality, a Cox proportional hazards regression model with MMA and smooth curve fitting (penalized spline approach) was used. The fully adjusted smooth curve fitting revealed a correlation between MMA and all-cause (Figure 2A), CVD- (Figure 2B), and cancer-related mortality (Figure 2C). After multivariate adjustment, a linear relationship was demonstrated (*P* < 0.001); each one-unit increase in the natural log-transformed MMA level was correlated with an increased all-cause mortality risk by 2.683 times, increased CVD mortality risk by 2.062 times, and increased cancer mortality risk by 4.515 times (Table 2). We performed a competing-risk model in Supplementary Figure 1. The results showed that higher serum MMA levels were significantly associated with higher CVD, cancer, and other causes (non-cancer or non-CVD) mortality.

### Stratification analyses

In the stratified analyses of the dose-response association between MMA levels and elevated all-cause mortality, we found that the subgroups of age >60 years, female sex, White race, BMI < 30 kg/m^2^, current smokers, vitamin B12 ≥ 400 pmol/L, and inactive physical activity were similar to our main results (Table 3). We also investigated the associations between MMA, CVD, and cancer-associated mortality. The CVD mortality results showed that the subgroups of male sex, White race, BMI ≥ 30 kg/m^2^, current smokers, and vitamin B12 < 400 pmol/L were similar to our main results (Supplementary Table 1). The results of cancer mortality showed that the subgroups of age ≤ 60 years, White race, BMI ≥ 30 kg/m^2^, current smokers (no and yes), and inactive physical activity were similar to our main results (Supplementary Table 2).

**TABLE 3:** Stratified analyses of the associations (hazard ratios, 95% confidence intervals [CIs]) between serum methylmalonic acid (MMA) concentrations and all-cause mortality among diabetes in National Health and Nutrition Examination Survey (NHANES).

	Serum MMA concentrations, nmol/L				
	<120	120–175	175–250	≥250	*P*-trend
Age, years*					
≤60	1.000 (ref.)	1.543 (0.763–3.121)	1.149 (0.418–3.158)	3.331 (1.206–9.198)	0.118
>60	1.000 (ref.)	1.240 (0.782–1.967)	2.186 (1.317–3.627)	2.186 (1.636–4.398)	0.000
Sex*					
Male	1.000 (ref.)	1.258 (0.803–1.973)	1.561 (0.915–2.662)	1.901 (1.102–3.277)	0.119
Female	1.000 (ref.)	1.013 (0.481–2.133)	2.030 (0.857–4.806)	3.154 (1.434–6.935)	0.004
Race/ethnicity*					
White	1.000 (ref.)	1.378 (0.868–2.185)	1.787 (1.054–3.030)	2.385 (1.440–3.951)	0.004
Non-White	1.000 (ref.)	1.060 (0.514–2.186)	2.031 (0.900–4.579)	2.513 (0.860–7.344)	0.173
BMI, kg/m^2^*					
<30	1.000 (ref.)	1.361 (0.823–2.249)	1.523 (0.825–2.812)	3.141 (1.754–5.623)	0.001
≥30	1.000 (ref.)	1.179 (0.636–2.187	1.901 (0.957–3.776	1.744 (0.874–3.481)	0.194
Current smoker*					
Yes	1.000 (ref.)	1.465 (0.940–2.283)	2.314 (1.351–3.963)	4.812 (3.045–7.604)	0.000
No	1.000 (ref.)	2.136 (1.672–2.727)	3.610 (2.800–4.656)	6.089 (4.778–7.758)	0.000
Vitamin B12, pmol/L*					
<400	1.000 (ref.)	1.304 (0.756–2.249)	1.551 (0.756–2.895)	1.921 (1.053–3.506)	0.168
≥400	1.000 (ref.)	1.240 (0.714–2.152)	1.785 (0.915–3.481)	3.191 (1.606–6.340)	0.008
Physical activity*					
Vigorous or Moderate activity	1.000 (ref.)	1.121 (0.638–1.971)	1.231 (0.591–2.565)	1.937 (1.012–3.707)	0.187
Inactive	1.000 (ref.)	1.438 (0.858–2.410)	2.165 (1.214–3.862)	2.620 (1.449–4.739)	0.007

*HRs (95% CI) were assessed using weighted Cox proportional regression fully adjusted except for stratification factor.

### Sensitivity analysis

In the sensitivity analyses, the results remained largely unchanged. After further adjustment for creatinine, total cholesterol, HDL-cholesterol, and self-reported disease (hypertension and hypercholesterolemia), the findings did not markedly change, although the relationship between MMA levels and mortality was slightly attenuated (Supplementary Table 3). However, the results of CVD mortality were not significant, which could be due to the reduced power.

## Discussion

This is the first prospective analysis of serum MMA concentrations in individuals with diabetes and all-cause and cause-specific mortality risks. This study revealed that the serum MMA concentration markedly correlated with high all-cause, CVD, and cancer mortality. The correlation was independent of traditional risk factors, such as demographics, socioeconomic status, education, ethnicity, smoking, comorbidity, BMI, and laboratory parameters. In relation to individuals with serum MMA concentrations <120 nmol/L, serum MMA of 120–175 nmol/L at baseline was associated with an HR point estimate of 1.228, serum MMA of 175–250 nmol/L correlated with an HR point estimate of 1.698, and serum MMA ≥ 250 nmol/L was associated with an HR point estimate of 2.117 for all-cause mortality. The correlation between MMA levels and mortality in patients with diabetes was slightly attenuated during further adjustment for laboratory parameters and self-reported disease.

Several epidemiological studies have shown that MMA is a potential risk factor for CVD and tumor progression. In relation to healthy control group, MMA concentrations in patients with myocardial infarction or heart failure markedly increased (16, 17). In contrast, another study comprising 300 patients with acute myocardial infarction showed no correlation of elevated MMA levels with increased risk of adverse outcomes during a median follow-up time of 45 months (18). These differences in outcomes may be due to variations in cohort study characteristics, sample sizes, and various confounding factors. Additionally, MMA has been reported to induce SOX4 and initiate transcriptional reprogramming, which can bestow aggressive characteristics to tumor cells (19). However, investigations on the prognostic role of MMA in cancer are lacking. Recently, an epidemiological study showed that elevated MMA levels were independently correlated with an increased risk of all-cause mortality in the general population (20). In the present study, our results revealed a non-linear correlation between MMA and all-cause mortality. Taken together, MMA might be vital for guiding improvements in health and longevity of the general and diabetes populations.

Circulating MMA levels partially depend on plasma vitamin B12 levels, renal function, and other factors (21–23). In this study, the correlation between MMA levels and all-cause and cause-specific mortality in patients with diabetes remained significant after adjusting for creatinine and vitamin B12 levels, indicating that the association was independent of traditional risk factors. A previous study has reported that only 22% of the variations in MMA levels were explained by vitamin B12, age, estimated glomerular filtration rate, and sex (20). Furthermore, Vogiatzoglou et al. (7) reported that 16% of variations in MMA levels were explained by plasma creatinine, vitamin B12, and sex. In addition, the results of the study by Vogiatzoglou et al. (7) could not identify other factors that substantially influence plasma MMA.

The pathophysiology of the relationship between MMA and mortality has not been completely elucidated. Mitochondria are critical regulatory centers for multiple cellular processes (24). Mitochondrial damage leads to ROS overproduction, resulting in oxidative stress, and apoptosis (25). Mitochondrial toxicity of MMA has been assessed both *in vitro* and *in vivo* (26). Furthermore, MMA induces apoptosis and oxidative stress (27–29). Mitochondrial dysfunction, characterized by impaired bioenergy and redox imbalance, is a prominent feature of several chronic diseases, such as CVD and diabetes, which markedly influence health, disease occurrence and progression, and life span (30). We further found that elevated MMA levels were primarily correlated with CVD and cancer-related mortality in patients with diabetes. Mitochondrial damage leads to ROS overproduction, which may lead to tumor cell initiation, progression, and growth by activating redox-responsive signaling cascades (31) and is one of the major causes of CVD (32). As this was not a mechanistic study, we postulated the potential underlying pathophysiological mechanisms of this relationship. Future studies should assess the role of MMA in diabetes.

The main strengths of this study included the use of a nationally representative sample which allows generalization of findings to the general population of the United States, a long-term follow-up period, and low rates of unmatched records in the NHANES Linked Mortality File. Moreover, using comprehensive data from NHANES, we controlled for potential confounding effects of various demographic, lifestyle, socioeconomic, and dietary factors.

This study has some limitations. First, as an observational study, the ability to establish cause-effect associations among and between variables is limited. Second, since the NHANES database was used, self-reported variables could not be excluded, leading to a reporting bias, and the results can only be extrapolated to the population of the United States. There is a need to verify the generalizability of these findings to other populations. Third, the small sample size was insufficient to fully assess the relationship between MMA and other causes of death. Last, even though we considered ≥10 potential confounding factors, such as personal variables, history of chronic conditions, lifestyle factors, and residual and unmeasured confounding factors might have affected the relationship.

## Conclusion

In conclusion, higher circulating MMA levels among individuals with diabetes were correlated with increased all-cause mortality, cancer mortality, and CVD mortality risk.

## Data availability statement

The original contributions presented in this study are included in the article/Supplementary material, further inquiries can be directed to the corresponding authors.

## Ethics statement

The survey protocol was approved by the Research Ethics Review Board of the National Center for Health Statistics (https://www.cdc.gov/nchs/nhanes/irba98.htm) and NHANES has obtained written informed consent from all participants.

## Author contributions

JX and WC designed the study protocol. JW and YT conducted the research and performed the data analysis under the close supervision of JX. YL provided the language help. JW wrote the manuscript and proofread by YT. All authors contributed to the article and approved the submitted version.

## Footnotes

1
http://www.R-project.org

2
www.empowerstats.com

## Abbreviations

BMI: body mass index
CHD: coronary heart disease
CVD: cardiovascular disease
DBP: diastolic blood pressure
eGFR: estimated glomerular filtration rate
HbA1c: glycated hemoglobin
HCys: homocysteine
HR: hazard ratio
MMA: methylmalonic acid
NHANES: National Health and Nutrition Examination Survey
SBP: systolic blood pressure.
