# Supplementary material for: Correlations between circulating methylmalonic acid levels and all-cause and cause-specific mortality among patients with diabetes
Source: Front Nutr. 2022 Nov 29;9:974938. doi: 10.3389/fnut.2022.974938 (PMC9745031; doi:10.3389/fnut.2022.974938)
Supplement: Supplementary file 1 [file Table_1.DOCX]

| **Table S1. Stratified analyses of the associations (hazard ratios, 95% CIs) between serum MMA concentrations and CVD mortality among diabetes in NHANES.** | | | | | |
| --- | --- | --- | --- | --- | --- |
|  | **Serum MMA concentrations, nmol/L** | | | | |
|  | **<120** | **120-175** | **175-250** | **≥250** | **P-trend** |
| **Age, years** |  |  |  |  |  |
| ≤60 | 1.000(ref.) | 7.418(0.703-78.298) | 0.160(0.000-7517.187) | 7.213(0.236-220.128) | 0.350 |
| >60 | 1.000(ref.) | 1.920(0.823-4.477) | 1.359(0.464-3.976) | 3.192(1.275-7.995) | 0.066 |
| **Sex** |  |  |  |  |  |
| Male | 1.000(ref.) | 2.693(1.071-6.775) | 0.561(0.131-2.404) | 2.436(0.820-7.239) | 0.019 |
| Female | 1.000(ref.) | 1.533(0.263-8.944) | 4.575(0.524-39.923) | 4.344(0.584-32.333) | 0.351 |
| **Race/ethnicity** |  |  |  |  |  |
| White | 1.000(ref.) | 3.252(1.201-8.807) | 1.457(0.404-5.263) | 3.799(1.287-11.215) | 0.037 |
| Non-White | 1.000(ref.) | 5.910(1.089-32.082 | 2.594(0.264-25.494 | 4.309(0.343-54.083) | 0.231 |
| **BMI,kg/m^2^** |  |  |  |  |  |
| <30 | 1.000(ref.) | 2.039(0.644-6.459) | 1.777(0.439-7.184) | 3.946(1.109-14.043) | 0.160 |
| ≥30 | 1.000(ref.) | 2.044(0.658-6.348) | 0.224(0.039-1.272) | 0.809(0.200-3.273) | 0.030 |
| **Current smoker** |  |  |  |  |  |
| Yes | 1.000(ref.) | 2.457(0.839-7.197) | 5.422(1.716-17.128) | 9.931(3.430-28.757) | 0.000 |
| No | 1.000(ref.) | 2.136(1.367-3.335 | 3.409(2.132-5.451 | 6.255(4.024-9.723) | 0.000 |
| **Vitamin B12, pmol/L** |  |  |  |  |  |
| <400 | 1.000(ref.) | 4.386(1.221-15.759 | 0.558(0.086-3.632 | 3.414(0.844-13.812) | 0.010 |
| ≥400 | 1.000(ref.) | 1.378(0.427-4.443) | 1.544(0.334-7.144) | 3.936(0.782-19.817) | 0.420 |
| **Physical activity** |  |  |  |  |  |
| Vigorous or Moderate activity | 1.000(ref.) | 2.319(0.743-7.238) | 0.550(0.091-3.324) | 3.052(0.835-11.160) | 0.088 |
| Inactive | 1.000(ref.) | 2.209(0.750-6.505) | 1.070(0.278-4.121) | 1.971(0.561-6.922) | 0.345 |
| *HRs(95%CI) were assessed using weighted Cox proportional regression fully adjusted except for stratification factor. | | | | | |
