# Supplementary material for: Correlations between circulating methylmalonic acid levels and all-cause and cause-specific mortality among patients with diabetes
Source: Front Nutr. 2022 Nov 29;9:974938. doi: 10.3389/fnut.2022.974938 (PMC9745031; doi:10.3389/fnut.2022.974938)
Supplement: Supplementary file 2 [file Table_2.DOCX]

| **Table S2. Stratified analyses of the associations (hazard ratios, 95% CIs) between serum MMA concentrations and** **cancer mortality among diabetes in NHANES.** | | | | | |
| --- | --- | --- | --- | --- | --- |
|  | **Serum MMA concentrations, nmol/L** | | | | |
|  | **<120** | **120-175** | **175-250** | **≥250** | **P-trend** |
| **Age, years** |  |  |  |  |  |
| ≤60 | 1.000(ref.) | 1.209(0.120-12.159) | 2.610(0.165-41.260) | 24.080(2.117-273.960) | 0.035 |
| >60 | 1.000(ref.) | 1.222(0.367-4.072) | 2.395(0.673-8.526) | 3.932(1.129-13.693) | 0.073 |
| **Sex** |  |  |  |  |  |
| Male | 1.000(ref.) | 1.126(0.355-3.574) | 1.497(0.404-5.554) | 3.440(0.903-13.113) | 0.221 |
| Female | 1.000(ref.) | 1.617(0.207-12.644) | 8.773(1.082-71.099) | 6.313(0.765-52.107) | 0.082 |
| **Race/ethnicity** |  |  |  |  |  |
| White | 1.000(ref.) | 1.254(0.338-4.650) | 2.410(0.594-9.788) | 5.829(1.539-22.070) | 0.013 |
| Non-White | 1.000(ref.) | 1.181(0.266-5.231) | 2.031(0.372-11.102) | 9.176(1.415-59.490) | 0.114 |
| **BMI,kg/m^2^** |  |  |  |  |  |
| <30 | 1.000(ref.) | 0.891(0.269-2.952) | 0.883(0.195-3.991) | 3.009(0.829-10.925) | 0.120 |
| ≥30 | 1.000(ref.) | 6.941(0.658-73.186) | 91.645(6.237-1346.538) | 47.877(3.269-701.225) | 0.005 |
| **Current smoker** |  |  |  |  |  |
| Yes | 1.000(ref.) | 0.881(0.334-2.322) | 1.932(0.657-5.678) | 3.501(1.367-8.962) | 0.023 |
| No | 1.000(ref.) | 2.357(1.296-4.287) | 2.690(1.385-5.227) | 3.914(2.065-7.421) | 0.000 |
| **Vitamin B12, pmol/L** |  |  |  |  |  |
| <400 | 1.000(ref.) | 1.685(0.519-5.467) | 2.018(0.553-7.362) | 3.949(1.123-13.891) | 0.159 |
| ≥400 | 1.000(ref.) | 0.806(0.100-6.459) | 1.562(0.167-14.645) | 7.890(1.264-49.245) | 0.079 |
| **Physical activity** |  |  |  |  |  |
| Vigorous or Moderate activity | 1.000(ref.) | 1.339(0.069-26.104) | 3.312(0.118-92.674) | 2.511(0.121-52.127) | 0.803 |
| Inactive | 1.000(ref.) | 0.952(0.312-2.905) | 1.802(0.553-5.872) | 5.479(1.643-18.274) | 0.009 |
| *HRs(95%CI) were assessed using weighted Cox proportional regression fully adjusted except for stratification factor. | | | | | |
