# Supplementary material for: Correlations between circulating methylmalonic acid levels and all-cause and cause-specific mortality among patients with diabetes
Source: Front Nutr. 2022 Nov 29;9:974938. doi: 10.3389/fnut.2022.974938 (PMC9745031; doi:10.3389/fnut.2022.974938)
Supplement: Supplementary file 3 [file Table_3.DOCX]

| **Table S3. HR (95% CIs) for all-cause and cause-specific mortality according to serum MMA concentrations among diabetes with further adjustment of Creatinine, blood lipids, and blood pressure in NHANES.** | | | | | |
| --- | --- | --- | --- | --- | --- |
| Causes of death | Serum MMA concentrations, nmol/L | | | |  |
|  | <120 | 120-175 | 175-250 | ≥250 | p trend |
| **All causes** **mortality** |  |  |  |  |  |
| Model 1 | 1.000(ref.) | 1.188(0.813-1.737) | 1.552(0.994-2.423) | 1.941(1.247-3.021) | 0.017 |
| Model 2 | 1.000(ref.) | 2.272(1.051-4.910) | 0.949(0.330-2.732) | 2.282(0.928-5.610) | 0.044 |
| Model 3 | 1.000(ref.) | 1.202(0.457-3.163) | 1.753(0.605-5.077) | 4.179(1.482-11.789) | 0.017 |
| **CVD mortality** |  |  |  |  |  |
| Model 1 | 1.000(ref.) | 1.168(0.798-1.710) | 1.526(0.976-2.388) | 1.901(1.219-2.965) | 0.022 |
| Model 2 | 1.000(ref.) | 2.198(1.015-4.760) | 0.899(0.309-2.611) | 2.191(0.885-5.424) | 0.048 |
| Model 3 | 1.000(ref.) | 1.412(0.532-3.748 | 2.083(0.715-6.066) | 3.423(1.145-10.236) | 0.125 |
| **Cancer mortality** |  |  |  |  |  |
| Model 1 | 1.000(ref.) | 1.240(0.807-1.906) | 1.587(0.961-2.619) | 1.965(1.190-3.244) | 0.045 |
| Model 2 | 1.000(ref.) | 2.338(1.024-5.335) | 0.749(0.226-2.485) | 1.811(0.668-4.907) | 0.043 |
| Model 3 | 1.000(ref.) | 1.077(0.359-3.231 | 1.239(0.336-4.575 | 3.968(1.162-13.551) | 0.042 |
| **Model 1**: adjusted for age (continuous), sex (male, or female), and race/ethnicity (non-Hispanic white, non-Hispanic black, Mexican American, or other), BMI (<25.0, 25.0-30, or ≥30 kg/m2 ), education level (less than high school, high school or equivalent, or college or above), family income-poverty ratio (≤1.0, 1.0-3.0, or >3.0), Alcohol drinks (continuous), smoking status (never smoker, ever smoker, or current smoker), and Physical activity (vigorous activity, moderate activity, or Inactive), Vitamin B12 ( pmol/L, continuous), diabetes medication use (no insulin or pills, only diabetes pills, only insulin, or pills and insulin), Creatinine (umol/L, continuous);  **Model 2:** model1 + total cholesterol (mmol/L, continuous), and HDL-cholesterol (mmol/L, continuous);  **Model 3:** model1 + self-reported hypertension, and hypercholesterolemia (yes, or no). | | | | | |
