# Supplementary material for: Correlations between circulating methylmalonic acid levels and all-cause and cause-specific mortality among patients with diabetes
Source: Front Nutr. 2022 Nov 29;9:974938. doi: 10.3389/fnut.2022.974938 (PMC9745031; doi:10.3389/fnut.2022.974938)
Supplement: Supplementary file 4 [file Data_Sheet_1.PDF]

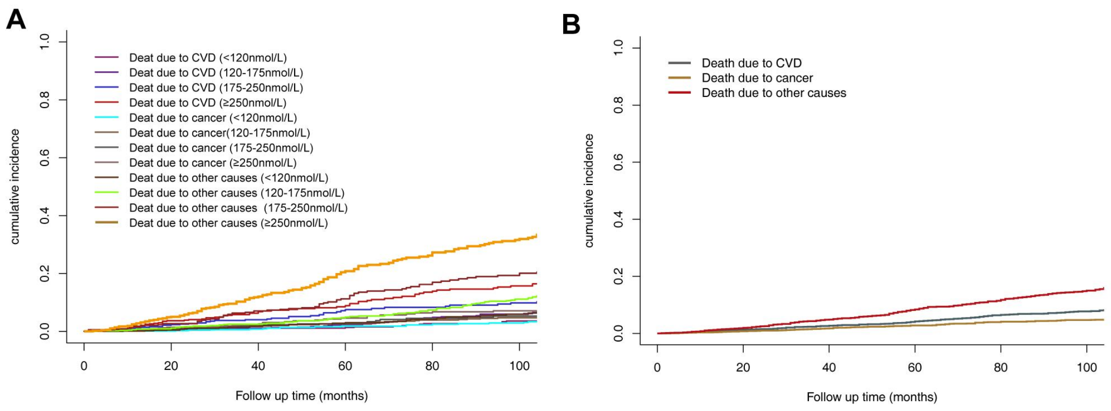

Figure S1: Competing risk analyses for mortality. (A) Competing risk regression for CVD, cancer, and other causes mortality with different MMA levels. (B) Competing risk regression for CVD, cancer, and other causes mortality.
